# Higher expression of SATB2 in hepatocellular carcinoma of African Americans determines more aggressive phenotypes than those of Caucasian Americans

**DOI:** 10.1111/jcmm.14652

**Published:** 2019-10-11

**Authors:** Wei Yu, Sanjit K. Roy, Yiming Ma, Thomas A. LaVeist, Sharmila Shankar, Rakesh K. Srivastava

**Affiliations:** ^1^ Kansas City VA Medical Center Kansas City MO USA; ^2^ Stanley S. Scott Cancer Center School of Medicine Louisiana State University Health‐New Orleans New Orleans LA USA; ^3^ Department of Health Policy and Management Tulane University School of Public Health and Tropical Medicine New Orleans LA USA; ^4^ Department of Genetics Louisiana State University Health Sciences Center‐New Orleans New Orleans LA USA

**Keywords:** cancer stem cells, c‐Myc, hepatocellular carcinoma, KLF4, miR‐34a, OCT4, pluripotency, SATB2, self‐renewal, SOX2

## Abstract

In the United States, Hepatocellular Carcinoma (HCC) incidence has tripled over the past two decades. The disease has disproportionately affected minority and disadvantaged populations. The purpose of this study was to examine the expression of SATB2 gene in HCC cells derived from African Americans (AA) and Caucasian Americans (CA) and assess its oncogenic potential by measuring cell viability, spheroid formation, epithelial‐mesenchymal transition (EMT), stem cell markers and pluripotency maintaining factors in cancer stem cells (CSCs). We compared the expression of SATB2 in human primary hepatocytes, HCC cells derived from AA and CA, and HCC CSCs. Hepatocellular carcinoma cells derived from AA expressed the higher level of SATB2 than those from CA. By comparison, normal human hepatocytes did not express SATB2. Higher expression of SATB2 in HCC cells from AA was associated with greater growth rate, cell viability, colony formation and EMT characteristics than those from CA. Knockout of SATB2 in CSCs by Crispr/Cas9 technique significantly inhibited the expression of SATB2 gene, stem cell markers (CD24, CD44 and CD133), pluripotency maintaining factors (c‐Myc, KLF4, SOX2 and OCT4), and EMT compared with non‐targeting control group. The expression of SATB2 was negatively correlated with miR34a. SATB2 rescued the miR‐34a‐mediated inhibition of CSC's viability. These data suggest that SATB2 is an oncogenic factor, and its higher expression may explain the disparity in HCC outcomes among AA.

## 1 INTRODUCTION

Hepatocellular carcinoma (HCC) is a leading cause of cancer‐related mortality and morbidity worldwide.^1^ In the United States, the incidence of HCC has more than quadrupled and became the fastest growing cause of cancer‐related deaths. According to the American Cancer Society, the HCC is expected to become the third leading cause of cancer‐related mortality by 2030.^2^, ^3^ Furthermore, the largest proportional increase in HCC incidence was reported among Hispanics and blacks.^4^, ^5^ In addition to chronic hepatitis B virus (HBV), chronic hepatitis C virus (HCV), alcoholic liver disease and non‐alcoholic steatohepatitis, social determinants are driving the epidemic at the population level.^6^ Furthermore, the disease has disproportionately affected all US racial/ethnic minority and disadvantaged populations.^7^ There is an unmet need to fully understand the existing disparities in HCC epidemiology so that the awareness in screening, surveillance and treatment opportunities can be improved to overcome the disparities in minority populations.

SATB2 (special AT‐rich binding protein‐2), a transcription factor and epigenetic regulator,^8^, ^9^ which regulates gene expression.^8^, ^10^, ^11^, ^12^, ^13^ It can modulate the expression of pluripotency maintaining factors and stem cell markers.^14^, ^15^, ^16^ Mice lacking SATB2 gene are defective in bone development and osteoblast differentiation.^11^ SATB2 has been associated with craniofacial patterning, osteoblast differentiation and cortical neurons development.^8^, ^11^, ^12^, ^13^, ^17^ SATB2 is highly expressed in embryonic stem cells and progenitor cells, whereas its expression is low or absent in human normal tissues. Our recent data showed that SATB2 gene can transform normal epithelial cells to cancer stem‐like cells in pancreatic, colorectal and breast cancer models, and the expression of SATB2 was significantly higher in cancer tissues^14^, ^15^, ^18^ compared with normal counterparts. We have also demonstrated that chronic ethanol exposure and reactive oxygen species can induce SATB2 in pancreatic ductal epithelial cells, suggesting it can be regulated by stress and inflammation.^19^ However, the mechanisms of differential expression and regulation of SATB2 in HCC of AA and CA have never been examined.

The main goal of the paper is to examine the role of SATB2 in HCC and assess whether it is differentially expressed in HCC derived from African Americans (AA) and Caucasian Americans (CA). Our data demonstrate that (a) SATB2 is highly expressed in HCC cells derived from AA compared with those from CA, (b) inhibition of SATB2 by Crispr/Cas9 techniques in HCC cells from AA and CA origins attenuates cell proliferation, colony formation and epithelial‐mesenchymal transition (EMT), (c) SATB2 regulates CSC characteristics by modulating the expression of stem cell markers (CD133, CD44 and CD24), pluripotency maintaining factors (c‐Myc, KLF4, SOX2 and OCT4), and EMT transcription factors (Snail, Slug and Zaeb1), (d) SATB2 rescued the miR‐34a‐mediated inhibition of CSC's viability, and (e) silencing of SATB2 gene inhibits spheroid formation. These data suggest that SATB2 is an oncogenic factor and its expression, which is higher in HCC cells derived from AA, may explain the disparity in HCC outcomes among AA.

## 2 MATERIALS AND METHODS

### 2.1 Reagents

Antibody against SATB2 was purchased from Abcam. Antibody against GAPDH was purchased from Cell Signalling Technology. Pierce™ Enhanced Chemiluminescence (ECL) Plus Western blotting substrate and Pierce™ BCA Protein Assay Kit were purchased from Thermo Fisher. A set of Crisper/cas9 lentiviral plasmids against human SATB2 gene and non‐targeting control (NTC) were purchased from GeneCopoeia™. BD Matrigel™ was purchased from BD Bioscience. TRIzol was purchased from Invitrogen. CellTiter‐Glo® Luminescent Cell Viability Assay and Luciferase assay kits were purchased from Promega Corporation. All other chemicals were purchased from Sigma‐Aldrich.

### 2.2 Cell culture

Hep3B, HepG2 and HEK293 cells were purchased from American Type Culture Collection (ATCC) and were authenticated by the vendor using short tandem repeat (STR) profiling. Human normal hepatocytes were obtained from the University of Kansas Liver Center under IRB approved protocol. Hepatocellular carcinoma cell lines were grown in Dulbecco's Modified Eagle's Medium (DMEM) with 10% Foetal Bovine Serum (HyClone) with antibiotics. CD24+/CD44+/CD133+ human liver cancer stem cells (CSCs) were isolated from primary HCC (obtained from Celprogen) and grown in well‐defined stem cell culture medium as per the supplier's instructions. All cells were used within 6 months of continuous passage and checked for the absence of *Mycoplasma* using detection kit (Lonza).

### 2.3 Lentiviral particle production and transduction

The protocol for lentivirus production and transduction have been described elsewhere.^20^ Briefly, 293T cells were transfected with 4 µg of plasmid and 4 µg of the lentiviral vectors using Lipofectamine‐3000 according to the manufacturer's protocol (Invitrogen). PEG‐it virus precipitation solution (SBI System Biosciences) was added to supernatant and ultracentrifugation was performed to collect concentrated viral particles. Hepatocellular carcinoma cells and CSCs were transduced with lentiviral particles with 6 μg/mL polybreme (Invitrogen).

### 2.4 Colony formation assay

For colony formation assays, HCC cells or CSCs were seeded at a low density into 6‐well plates for about 3 weeks.^21^ After incubation, colonies were fixed with methanol, stained with 0.5% crystal violet and counted under a microscope.

### 2.5 Spheroid assay

Spheroid formation assays were performed as described elsewhere.^20^, ^22^ Briefly, HCC cells or CSCs at 100‐500 cells/mL density were plated in ultra‐low attachment plates. The spheroids formed in suspension were collected after 10 days of incubation.

### 2.6 Transwell migration assay

Transwell migration assay was performed as we described elsewhere.^20^, ^22^ In brief, HCC cells or CSCs (1 × 10^5^) were plated in the top chamber onto the non‐coated membrane (24‐well insert; pore size, 8 mm; BD Biosciences) and allowed to migrate in the lower chamber towards the serum‐containing medium. After 24 hours of incubation, cells were fixed with methanol, stained with crystal violet and counted.

### 2.7 Transwell invasion assay

Transwell invasion assay was performed using the Boyden chambers as we described elsewhere.^20^, ^21^ Briefly, the upper side of the filters was coated with Matrigel 1 mg/mL. Hepatocellular carcinoma cells or CSCs (1 × 10^5^) were seeded onto the layer of Matrigel in Transwell chambers (BD Biosciences). Cells were plated in medium without serum, and medium supplemented with FBS was used as a chemoattractant in the lower chamber. After 24 hours of incubation, unmigrated cells on the upper side of the filters were mechanically removed, whereas those invaded to the bottom chamber were fixed with methanol, stained with crystal violet and counted.

### 2.8 Western blot analysis

The Western blot analysis was performed as we described earlier.^23^ In brief, cells were washed once with ice‐cold PBS and suspended in RIPA lysis buffer (10 mM Tris‐HCl, pH 7.4, 140 mM NaCl, 1% Triton X‐100, 1% Sodium deoxycholate, 0.1% SDS, 1 mM Na_3_VO_4_, 1 mM NaF and 1 mM EDTA) added with a cocktail of protease inhibitors. Lysis was performed on ice for 30 minutes, and the samples were centrifuged at 17 000 *g* for 30 minutes. The concentration of the crude protein was measured with a bicinchoninic acid (BCA) Protein Assay Kit (Thermo Fisher Scientific). Equal amounts of cell lysates were loaded and separated by SDS‐PAGE, and gels were blotted to polyvinylidene difluoride membranes. Membranes with blotted proteins were incubated with primary antibodies, washed and incubated with peroxidase‐conjugated secondary antibodies. Reactive proteins were revealed with an enhanced chemiluminescent detection system and visualized on the imaging system.

### 2.9 Luciferase Reporter Assay

SATB2 as a MiR‐34a target was predicted using TargetScan (http://www.targetscan.org/), and mirDB (http://mirdb.org/). SATB2 promoter contains miR34a binding site. CSCs (3 × 10^4^/well) were seeded into 24‐well plates followed by transfection with the Renilla Luciferase pRL‐TK plasmid plus the Recombinant Firefly Luciferase pGL3 Reporters containing 3′ UTR region of human SATB2 in combination with PremiR‐34a and NTC. After transfection, cells were collected and lysed. Luciferase and Renilla signals were measured using a Dual‐Luciferase Reporter Assay Kit (Promega) according to the manufacturer's instructions.

### 2.10 Quantitative real‐time PCR

Total RNA was extracted with TRIzol reagent (Invitrogen), and the cDNA was generated by the Reverse Transcription System (Promega) in a 20 μL reaction containing 1 μg of total RNA. A 0.5 μL aliquot of cDNA was amplified by Fast SYBR Green PCR Master Mix (Applied Biosystems) in each 20 μL reaction. PCR reactions were run on the ABI 7900 Fast Real‐Time PCR system (Applied Biosystems).

### 2.11 Statistical analysis

All analyses were performed using GraphPad Prism Software (GrafPad Software, Inc). Statistical differences between groups were analysed using the Student *t* test or Analysis of Variance (ANOVA) followed by Bonferroni's multiple comparison tests. Significant differences among groups were calculated at *P* < .05. The mean ± SD or SE was calculated for each experimental group.

## 3 RESULTS

### 3.1 HCC cells derived from AA express higher levels of SATB2 than those derived from CA, and its expression is absent in human normal hepatocytes

**Figure 1:**
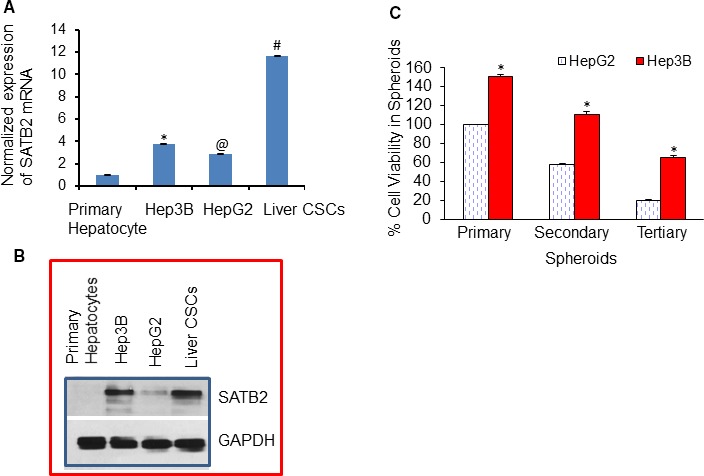
Expression of SATB2 in human normal hepatocytes, HCC cell lines and cancer stem cells, and measurement of cell viability in spheroids. A, Expression of SATB2 mRNA by qRT‐PCR. RNA was isolated from human primary hepatocytes, HCC cell lines (Hep3B and HepG2) and liver cancer stem cells (CSCs). The expression of SATB2 was measured by qRT‐PCR. GAPDH was used as an internal control. Data represent mean (n = 4) ± SD. *, @ and # = significantly different from primary hepatocytes (*P* < .05). B, Expression of SATB2 protein. Crude proteins from human primary hepatocytes, HCC cell lines (Hep3B and HepG2) and liver CSCs were isolated and the expression of SATB2 was measured by the Western blot analysis. GAPDH was used as a loading control. C, Comparison of cell viability in spheroids formed by HepG2 and Hep3B cells. Cells were seeded in an ultra‐low attachment six‐well plates. Spheroids formed after 1 wk were collected and dissociated. Cell viability in spheroids was calculated. For secondary spheroid formation, cells were reseeded, and spheroids were grown for another week. Spheroids were collected, and cell viability in spheroids was calculated. For tertiary spheroid formation, cells were reseeded, and spheroids were grown for another week. Spheroids were collected, and cell viability in spheroids was calculated. Data represent mean (n = 4) ± SD. * = significantly different from HepG2 cells (*P* < .05)

We first compared the expression of SATB2 in human normal primary hepatocytes and HCC cell lines (Hep3b and HepG2) and liver CSCs by qRT‐PCR and Western blot analysis. As shown in Figure 1A by qRT‐PCR, SATB2 is not expressed in human normal primary hepatocytes. However, it is highly expressed in Hep3b, HepG2 and liver CSCs. Interestingly, the expression of SATB2 was highest in liver CSCs. Hep3b cell lines derived from AA expressed higher levels of SATB2 than HepG2 cell lines derived from CA.

Since SATB2 mRNA was differentially expressed, we next measured the expression of SATB2 protein by the Western blot analysis. As shown in Figure 1B, SATB2 is not expressed in human normal primary hepatocytes, whereas the significant expression of SATB2 protein was noticed in Hep3b, HepG2 and liver CSCs. Liver CSCs expressed the highest level of SATB2 protein. Interestingly, Hep3B cells derived from AA expressed higher levels of SATB2 protein than HepG2 cells derived from CA. These data suggest that the expression of SATB2 is tightly regulated, and it is highly expressed in AA compared with CA.

Since spheroid formation is suspension is one of the characteristics of CSCs, we next compared the percentage of viable cells in spheroids formed by growing HpeG2 and Gep3B cells (Figure 1C). Cell viabilities in primary, secondary and tertiary spheroids formed by Hep3B cells were significantly higher than those in HepG2 spheroids. These data suggest that HCC cells isolated from AA tumours may possess an aggressive stem cell characteristic than those from CA.

### 3.2 Inhibition of SATB2 expression by Crispr/Cas9 technique in hepatocellular carcinoma suppresses cell viability, cell proliferation and colony formation

**Figure 2:**
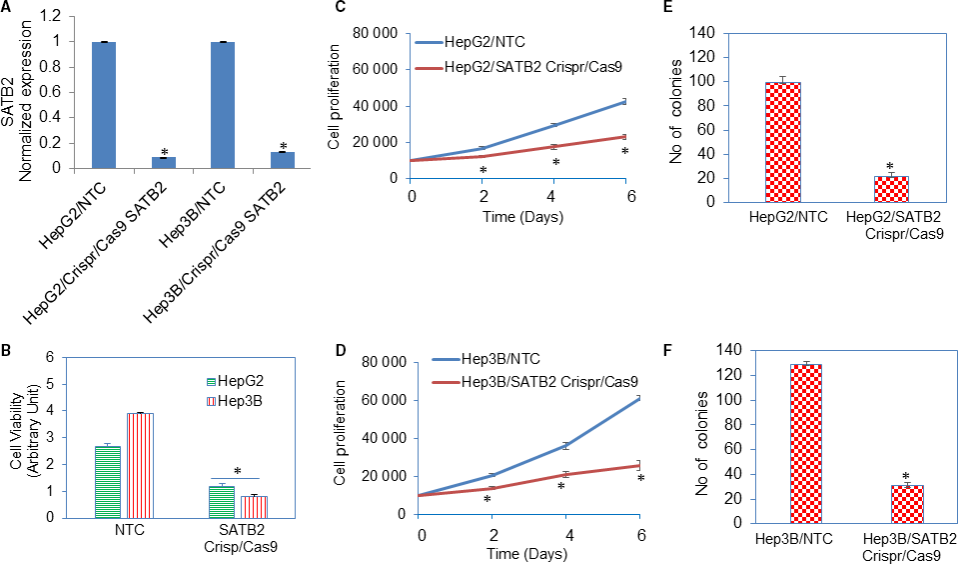
Inhibition of SATB2 expression suppresses cell viability and stem cell markers and pluripotency maintaining factors in HepG2 and Hep3B cells. A, HepG2 and Hep3B cells were transduced with lentiviral particles expressing either SATB2 Crispr/Cas9 or nor‐targeting control (NTC) from a pooled population by targeting four different sites (GeneCopoeia, Rockville, MD). RNA was extracted from cells, and qRT‐PCR analysis was performed to measure the expression of SATB2. Data represent mean ± SD (n = 4). * = significantly different from NTC group (*P* < .05). B, HepG2/NTC, Hep3B/NTC, HepG2/SATB2 Crispr/Cas9 and Hep3B/SATB2 Crispr/Cas9 cells were grown for 72 h, and cell viability was measured by XTT assay. Data represent mean ± SD (n = 4). * = significantly different from NTC group (*P* < .05). C, HepG2/NTC and HepG2/SATB2 Crispr/Cas9 cells were grown, and cell proliferation was measured on day‐2, day‐4 and day‐6. Data represent mean ± SD (n = 4). * = significantly different from NTC group (*P* < .05). D, Hep3B/NTC and Hep3B/SATB2 Crispr/Cas9 cells were grown, and cell proliferation was measured on day‐2, day‐4 and day‐6. Data represent mean ± SD (n = 4). * = significantly different from NTC group (*P* < .05). E, HepG2/NTC and HepG2/SATB2 Crispr/Cas9 cells were grown, and numbers of colonies formed on day‐21 were counted. Data represent mean ± SD (n = 4). * = significantly different from NTC group (*P* < .05). F, Hep3B/NTC and Hep3B/SATB2 Crispr/Cas9 cells were grown, and numbers of colonies formed on day‐21 were counted. Data represent mean ± SD (n = 4). * = significantly different from NTC group (*P* < .05)

SATB2 has been shown to regulate the oncogenesis in various cancers. We next sought to examine the effects of SATB2 on HCC by knocking down its expression by Crispr/Cas9 technique. HepG2 and Hep3B cells were transduced with lentiviral particles expressing either SATB2 Crispr/Cas9 or NTC from a pooled population by targeting four different sites (GeneCopoeia, Rockville, MD). RNA was extracted from cells and qRT‐PCR analysis was performed to measure the expression of SATB2. Inhibition of SATB2 expression in HepG2 and Hep3B cells by Crispr/Cas9 techniques suppressed the expression of SATB2 (Figure 2A). Furthermore, HepG2/SATB2 Crispr/Cas9 and Hep3B/SATB2 Crispr/Cas9 groups exhibited lower cell viability and cell proliferation than NTC groups (Figure 2B‐D).

The colony formation assay is a well‐established and accepted technique for characterizing the malignant growth property of cancer cells. We next employed the colony formation assay using HCC cell lines to demonstrate the tumour suppressive effects of SATB2. HepG2/SATB2 Crispr/Cas9 and Hep3B/SATB2 Crispr/Cas9 groups exhibited a lower number of colonies than NTC groups (Figure 2E,F). These data suggest that SATB2 gene is an oncogenic gene and its inhibition can result in HCC suppression.

### 3.3 Inhibition of SATB2 expression in HCC cells by Crispr/Cas9 technique suppresses epithelial‐mesenchymal transition

**Figure 3:**
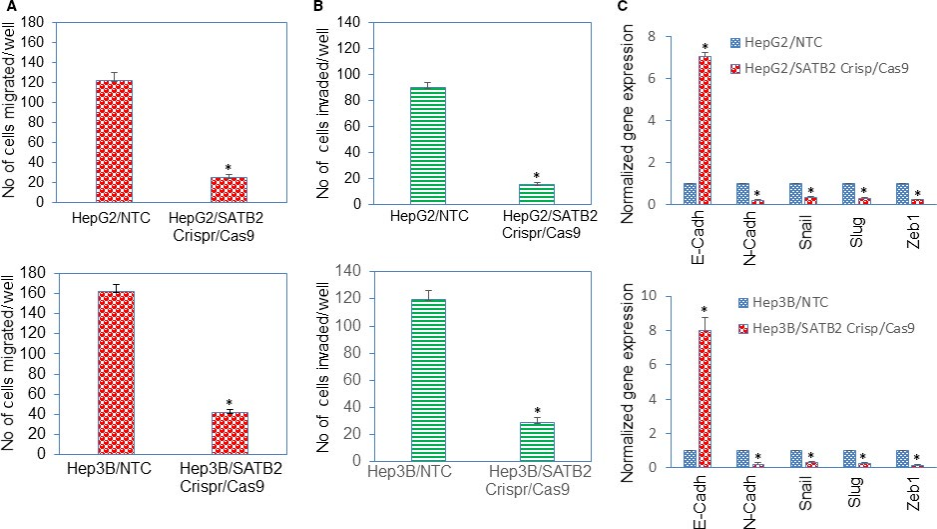
Inhibition of SATB2 expression suppresses cell migration, and invasion, and modulates the expression of EMT‐related gene expression in HepG2 and Hep3B cells. A, HepG2/NTC, Hep3B/NTC, HepG2/SATB2 Crispr/Cas9 and Hep3B/SATB2 Crispr/Cas9 cells were grown for 48 h. Cell migration assay was performed as described in Section 2. Data represent mean (n = 4) ± SD. * = significantly different from NTC control group, *P* < .05. B, HepG2/NTC, Hep3B/NTC, HepG2/SATB2 Crispr/Cas9 and Hep3B/SATB2 Crispr/Cas9 cells were grown for 48 h. Cell invasion assay was performed as described in Section 2. Data represent mean (n = 4) ± SD. * = significantly different from NTC control group, *P* < .05. C, HepG2/NTC, Hep3B/NTC, HepG2/SATB2 Crispr/Cas9 and Hep3B/SATB2 Crispr/Cas9 cells were grown. RNA was isolated and the expressions of E‐Cadherin, N‐Cadherin, Snail, Slug and Zeb1 were measured by qRT‐PCR analysis. Data represent mean (n = 4) ± SD. * = significantly different from NTC control group (*P* < .05). Gene expression of NTC control group was normalized to 1

An EMT is a biologic process by which polarized epithelial cells undergo multiple biochemical and morphological changes that enable them to assume mesenchymal cell phenotypes. These features include enhanced migratory capacity and invasiveness and increased production of ECM components.^24^ We next examined the ability of SATB2 to inhibit cell migration and invasion using transwell migration and invasion assay. Inhibition of SATB2 by Crispr/Cas9 technique inhibited cell migration and invasion in both HepG2 and Hep3B cell lines (Figure 3A,B). Furthermore, numbers of migrated and invaded Hep3B cells were higher than those of HepG2 cells.

Since SATB2 inhibited cell migration and invasion, we next examined the effects of SATB2 inhibition on genes (E‐cadherin, N‐Cadherin, Snail, Slug and Zeb1) which regulate EMT characteristics (Figure 3C). Inhibition of SATB2 resulted in upregulation of epithelial marker E‐cadherin and inhibition of mesenchymal marker N‐cadherin, and suppression of an EMT transcription factors Snail, Slug and Zeb1 in both HepG2 and Hep3B cells. These data suggest that inhibition of SATB2 expression can suppress EMT characteristics by modulating the expression of Cadherins and EMT‐related transcription factors.

### 3.4 Inhibition of SATB2 expression in cancer stem cells by Crispr/Cas9 technique suppresses cell viability in spheroids, stem cell markers and transcription factors

**Figure 4:**
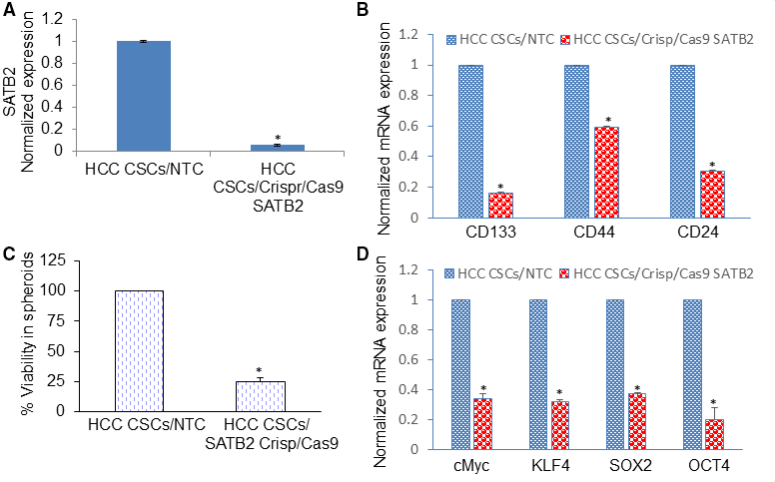
Inhibition of SATB2 expression suppresses cell viability in spheroids and stem cell markers and pluripotency maintaining factors in HepG2 and Hep3B cells. A, CSCs derived from primary HCC tissues were transduced with lentiviral particles expressing either SATB2 Crispr/Cas9 or non‐targeting control from a pooled population by targeting four different sites (GeneCopoeia, Rockville, MD). RNA was extracted from cells, and qRT‐PCR analysis was performed to measure the expression of SATB2. Data represent mean ± SD (n = 4). * = significantly different from NTC group (*P* < .05). B, CSCs/NTC and CSCs/SATB2 Crispr/Cas9 cells were grown for 6 d, and cell viability in spheroids was measured by XTT assay. Data represent mean ± SD (n = 4). * = significantly different from NTC group (*P* < .05). C and D, RNA was isolated from CSCs/NTC and CSCs/SATB2 Crispr/Cas9 cells. The qRT‐PCR analysis was performed to measure the expression of stem cell markers (CD133, CD44 and CD24) and pluripotency maintaining factors (c‐Myc, KLF4, SOX2 and OCT4). GAPDH was used as an internal control. Data represent mean ± SD (n = 4). * = significantly different from CSCs/NTC group, *P* < .05. Note that the expression of genes in the control group was normalized to 1

We have recently demonstrated the roles of SATB2 in malignant transformation and maintaining CSCs characteristics by regulating CSC markers and stem cell transcription factors in pancreatic, breast and colorectal cancers.^14^, ^15^, ^16^ Since SATB2 is a transcription factor and modulates stemness, we examined whether inhibition of SATB2 in CSCs by Crisp/Cas9 technique regulates cell viability, expression of stem cell markers and pluripotency maintaining factors. CSCs derived from primary HCC tissues were transduced with lentiviral particles expressing either SATB2 Crispr/Cas9 or NTC from a pooled population by targeting four different sites (GeneCopoeia, Rockville, MD). RNA was extracted from CSCs and qRT‐PCR analysis was performed to measure the expression of SATB2. CSCs/SATB2 Crispr/Cas9 group demonstrated lower expression of SATB2 than NTC group (Figure 4A). CSCs/SATB2 Crispr/Cas9 group exhibited significantly lower per cent cell viability in spheroids than those in NTC group (Figure 4B). Furthermore, spheroids formed by CSCs/SATB2 Crispr/Cas9 group were smaller than those formed by CSCs/NTC group (data not shown).

Since inhibition of SATB2 expression attenuated cell viability in CSC spheroids, we next measured the effects of inhibiting SATB2 expression on CSC markers and pluripotency maintaining factors. CSCs/SATB2 Crispr/Cas9 group demonstrated significantly lower levels of stem cell markers (CD24, CD44 and CD133) and pluripotency maintaining factors (c‐Myc, KLF4, SOX2 and OCT4) compared with NTC group (Figure 4C,D). These data suggest that SATB2 can regulate stem cell population by suppressing stem cell markers and pluripotency maintaining factors.

### 3.5 miR‐34a inhibits cell viability by targeting SATB2 in CSCs isolated from HCC

**Figure 5:**
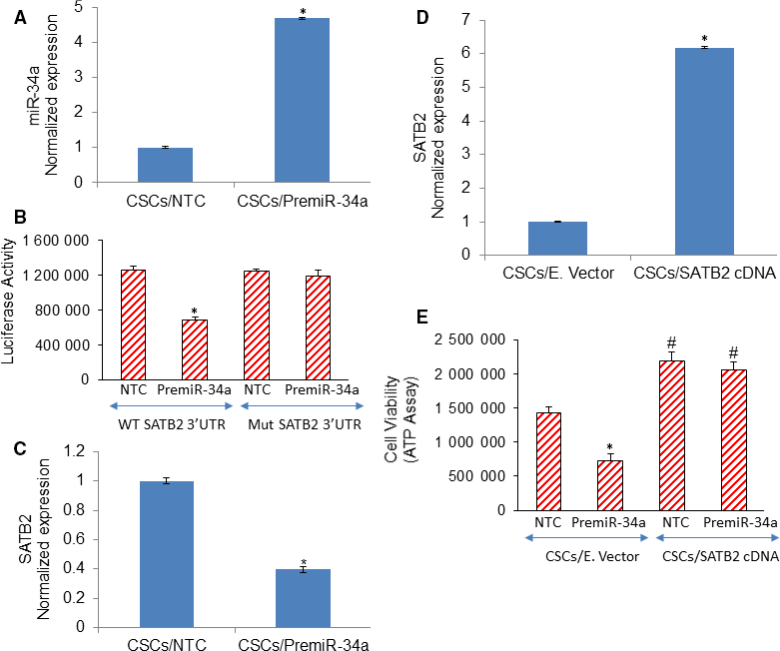
Mir‐34a inhibits CSC's viability by suppressing SATB2. A, Expression of miR‐34a. HCC CSCs was transduced with lentiviral particles expressing either PremiR‐34a or non‐targeting control (NTC). The expression of miR‐34a was measured by qRT‐PCR. Data represent mean ± SD (n = 4). * = significantly different from CSCs/NTC group, *P* < .05. B, miR‐34a inhibits SATB2 luciferase activity. The relative luciferase activities of STAB2‐wt, STAB2‐mut were measured using a Dual‐Luciferase Reporter Assay Kit following transduction with either premiR‐34a or NTC. Data represent mean ± SD (n = 4). * = significantly different from CSCs/NTC group, *P* < .05. C, miR‐34a inhibits SATB2 mRNA expression. HCC CSCs were transduced with lentiviral particles expressing either PremiR‐34a or NTC. The expression of miR‐34a was measured by qRT‐PCR. Data represent mean ± SD (n = 4). * = significantly different from CSCs/NTC group, *P* < .05. D, HCC CSCs were transduced with lentiviral particles expressing either SATB2 gene or empty vector. The expression of SATB2 was measured by qRT‐PCR. Data represent mean ± SD (n = 4). * = significantly different from CSCs/E. Vector group, *P* < .05. E, Cell Viability. CSCs/E. Vector and CSCs/SATB2 cDNA groups were transduced with either premiR‐34a or NTC. Cell viability was measured by CellTiter‐Glo® Luminescent Cell Viability Assay (Promega). Data represent mean ± SD (n = 4). * = significantly different from CSCs/NTC group, *P* < .05

The SATB2 is a direct target of miR34a.^25^, ^26^ We therefore examined whether miR34a regulates the expression of SATB2 in CSCs. Overexpression of Premir‐34a significantly enhanced the expression of miR‐34a compared with NTC group (Figure 5A). SATB2 3′‐UTR contained a conserved putative target site for miR‐34a. To validate the relationship between miR‐34a and SATB2, we examined the luciferase activity and the mRNA level of SATB2 in CSCs following transduction with PremiR‐34a. As shown in Figure 5B, PremiR‐34a inhibited the activity of Luciferase Reporter containing SATB2 3′‐UTR. In contrast, PremiR‐34a had no effect on Mut SATB2 promoter activity (lacking MiR‐34a binding site). Furthermore, overexpression of Premir‐34a in CSCs significantly inhibited the expression of SATB2 mRNA compared with NTC group (Figure 5C). Taken together, our data demonstrated that miR‐34a targets SATB2 and suppresses its expression in CSCs. These data also demonstrate a negative correlation between the expression levels of SATB2 and miR‐34a. We next examined whether overexpression of SATB2 rescued the miR‐34a‐mediated inhibition of CSC's viability. Cell viability was measured by ATP assay. Overexpression of SATB2 in CSCs enhanced SATB2 expression compared with empty vector group (Figure 5D). PremiR‐34a inhibited the cell viability in CSCs/E. Vector group. Overexpression of SATB2 rescued the premiR‐34a‐mediated inhibition of cell viability in CSCs (Figure 5E). These data suggest that SATB2 is a direct target of miR‐34a and can mediate its effects in HCC.

### 3.6 Knockdown of SATB2 in CSCs inhibits cell proliferation, colony formation and epithelial to mesenchymal transition

**Figure 6:**
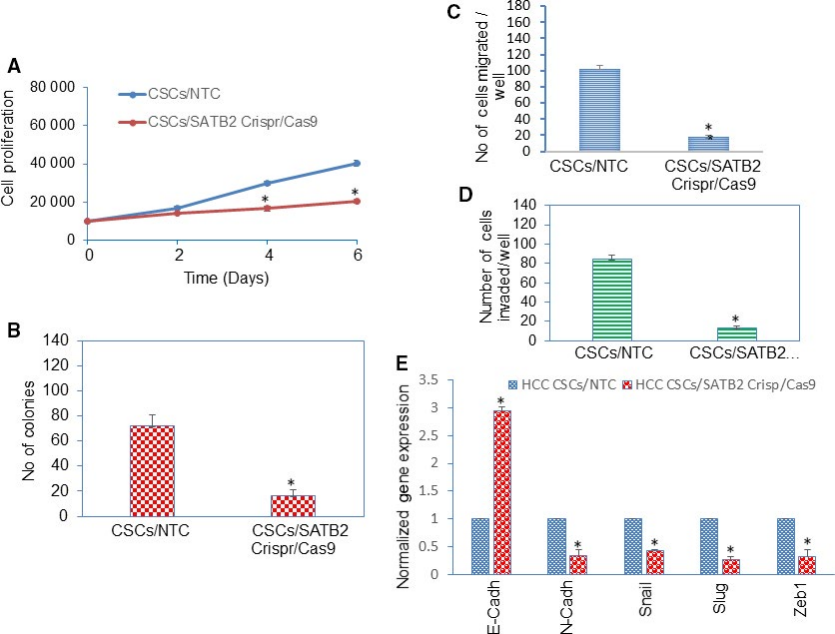
Inhibition of SATB2 expression suppresses cell proliferation, colony formation, cell migration and invasion, and modulates the expression of EMT‐related genes in CSCs isolated from HCC. A, CSCs/NTC and CSCs/SATB2 Crispr/Cas9 cells were grown, and CSC proliferation was measured on day‐2, day‐4 and day‐6. Data represent mean ± SD (n = 4). * = significantly different from CSCs/NTC group, *P* < .05. B, CSCs/NTC and CSCs/SATB2 Crispr/Cas9 cells were grown, and numbers of colonies formed on day‐21 were counted. Data represent mean ± SD (n = 4). * = significantly different from CSCs/NTC group, *P* < .05. C, Cell Migration. CSCs/NTC and CSCs/SATB2 Crispr/Cas9 cells were grown for 48 h. Cell migration assay was performed as described in Section 2. Data represent mean (n = 4) ± SD. * = significantly different from NTC group, *P* < .05. D, Cell Invasion. CSCs/NTC and CSCs/SATB2 Crispr/Cas9 cells were grown for 48 h. Cell invasion assay was performed as described in Section 2. Data represent mean (n = 4) ± SD. * = significantly different from NTC group, *P* < .05. E, CSCs/NTC and CSCs/SATB2 Crispr/Cas9 cells were grown. RNA was isolated and the expressions of E‐Cadherin, N‐Cadherin, Snail, Slug and Zeb1 were measured by qRT‐PCR analysis. Data represent mean (n = 4) ± SD. * = significantly different from NTC control group (*P* < .05). Gene expression of NTC control group was normalized to 1

The SATB2 is a transcription factor, regulates gene expression and controls stemness, cell survival, and differentiation.^27^, ^28^, ^29^, ^30^ We therefore examined whether inhibition of SATB2 by Crisp/Cas‐9 technique attenuates cell proliferation of CSCs. Cell proliferation of CSCs/NTC and CSCs/SATB2 Crispr/Cas9 groups were compared over the 6‐day period. CSCs/SATB2 Crispr/Cas9 group had lower rate of cell proliferation than CSCs/NTC group (Figure 6A). These data suggest that the knockdown of SATB2 in CSCs can suppress HCC growth. We next examined the effects of SATB2 on colony formation. The inhibition SATB2 expression by Crispr/Cas9 attenuated colony formation in CSCs/SATB2 Crispr/Cas9 group compared with CSCs/NTC group (Figure 6B). These data suggest that inhibition of SATB2 expression in CSCs can retard cell proliferation and colony formation.

Epithelial to mesenchymal transition (EMT) plays a key role in cell motility, invasion, migration and metastasis.^31^ Cells undergoing this transition modulate the expression of EMT‐related genes and show enhanced migration and invasion.^31^ We next examined whether inhibition of SATB2 by Crispr/Cas9 technique attenuates EMT characteristics in CSCs. CSCs were transduced with either NTC or SATB2 Crispr/Cas9 lentiviral particles as described above. SATB2 Crispr/Cas9 inhibited cell migration and invasion of CSCs (Figure 6C,D).

We next examined the effects of inhibiting SATB2 on the expression of EMT transcription factors and cadherins in CSCs by qRT‐PCR (Figure 6E). Transduction of CSCs with SATB2 Crispr/Cas9 lentiviral particles inhibited the expression of EMT‐related transcription factors (Slug, Snail and Zeb1) and N‐Cadherin, and enhanced the expression of E‐Cadherin. Overall, our data suggest that inhibition of SATB2 expression in CSCs can suppress EMT characteristics.

## 4 DISCUSSION

In the present study, we have demonstrated for the first time that SATB2 is differentially expressed in HCC cells derived from AA than those from CA. Hepatocellular carcinoma cells from AA origin expressed higher levels of SATB2 mRNA and protein and showed aggressive phenotypes than those from CA. The fact that SATB2 gene plays an oncogenic role in HCC was confirmed by Crispr/Cas9 technique. The inhibition of SATB2 by Crispr/Cas9 technique suppressed cell proliferation, spheroid formation, and colony formation not only in HCC but also in CSCs. Inhibition of SATB2 expression in HCC CSCs also suppressed CSC characteristics by inhibiting the expression of pluripotency maintaining factors (c‐Myc, OCT4, KLF4 and SOX2) and stem cell markers (CD24, CD44 and CD133). Since the SATB2 is highly expressed in the HCC cells, but nor in normal hepatocytes, it can be targeted for HCC biomarker and therapy.

The role of CSCs in HCC is well documented.^32^, ^33^, ^34^, ^35^, ^36^ The CSCs/tumour‐initiating cells can be responsible for cancer initiation, metastasis and resistance to therapy.^37^, ^38^, ^39^ Targeting of EpCAM+, or CD133+ CSCs have been shown to eradicate HCC.^34^, ^35^, ^40^, ^41^ The role of SATB2 in HCC is not well understood. SATB2 regulates transcriptional and remodels chromatin structure.^12^, ^42^, ^43^ Here, we have demonstrated that SATB2 can positively regulate the expression of stem cell markers CD24, CD44 and CD133 in HCC CSCs. Similarly, in other models, we have demonstrated that SATB2 shRNA can inhibit CSCs characteristics by suppressing the expression of stem cell markers and pluripotency maintaining factors.^14^, ^15^, ^16^, ^44^ Furthermore, we demonstrated that SATB2 can induce transformation of epithelial cells which gained the phenotypes of CSCs/progenitor cells by expressing stem cell markers (CD24, CD44 and CD133) and pluripotency maintaining factors (c‐Myc, OCT4, KLF4, Nanog and SOX2)..^14^, ^15^, ^16^ Inactivation of oncogene c‐Myc alone was able to reverse tumorigenesis in HCC as demonstrated by differentiation of tumour cells into hepatocytes and biliary cells forming bile duct structures. These structures exhibited a loss in the expression of α‐fetoprotein (tumour marker), and an increase in the expression of cytokeratin 8 (liver cell markers) and cytokeratin 19 (live stem cell marker).^45^ Oncogene inactivation caused cancer cells to differentiate into normal cellular lineages and tissue structures while retaining their latent potential to become cancerous and thereby showing a dormant stage of tumour.^45^ Our data suggest that deletion of SATB2 in HCC and CSCs can abolish stemness properties.

We have recently suggested the induction of SATB2 gene under conditions which create stressful environment. Specifically, SATB2 was induced by pancreatic ductal epithelial cells when they were exposed to chronic ethanol, a condition generates reactive oxygen species (ROS).^19^ Epidemiological data suggest that poor socio‐economic conditions, inadequate access to screening and treatment opportunities may create stressful and inflammatory environment,^46^, ^47^, ^48^, ^49^, ^50^, ^51^, ^52^ which may induce SATB2. This hypothesis needs to be tested in HCC disparities.

The liver is the first and most common organ to which cancers commonly metastasize and spread in patients. Recent studies have demonstrated the higher expression of SATB2 in the HCC tissues compared with adjacent normal tissues.^25^, ^26^, ^53^, ^54^ The commonly used markers for the HCC include metastatic carcinomas markers cytokeratins (CK7, CK19 and CK20), neuroendocrine markers (CD56, synaptophysin and chromogranin A) and tissue‐specific markers (SATB2, CDX2, TTF‐1, GCDFP‐15, mammaglobin, etc).^54^ microRNAs (miRNAs) regulate cancer cell progression and metastasis by modulating the expression of SATB2.^25^, ^26^ Our date showed a negative correlation between the expression levels of SATB2 and miR‐34a in CSCs. Overexpression of SATB2 rescued the miR‐34a mediated inhibition of cell viability in CSCs. Similarly, in recent studies of HCC, miR‐211 and miR34a levels inversely correlated with SATB2 levels and modulated cancer cell progression and metastasis.^25^, ^26^ Our data demonstrate that SATB2 can regulate cell migration and invasion by modulating EMT‐related genes (cadherins) and transcription factors (Snail, Slug and Zeb1). Furthermore, the invasion and migration capacities of HCC cells derived from AA were significantly higher than those of CA. Similarly, in the breast, pancreatic and colorectal cancer models we have demonstrated that SATB2 can positively regulate EMT characteristics by modulating the expressions of cadherins, vimentin and EMT‐related transcription factors.^14^, ^15^, ^16^ Overall, our studies suggest that SATB2 can promote EMT and metastasis in HCC.

In conclusions, our data demonstrate that (a) SATB2 is highly expressed in HCC cells derived from AA compared with those from CA, (b) inhibition of SATB2 in HCC cells derived from both AA and CA origins attenuates cell proliferation, colony formation, spheroid formation and EMT, and (c) SATB2 regulates CSC characteristics by modulating the expression of stem cell markers (CD133, CD44 and CD24), pluripotency maintaining factors (c‐Myc, KLF4, SOX2 and OCT4) and EMT transcription factors (Snail, Slug and Zeb1). These data suggest that SATB2 is an oncogenic factor and its expression, which is higher in HCC cells derived from AA, may explain the disparity in HCC outcomes among AA.

## CONFLICT OF INTEREST

All the authors have declared that no conflict of interest exists.

## AUTHOR CONTRIBUTIONS

WY, SKR and YM performed the experiments, analysed the data and wrote the manuscript. SS and RKS supervised activity planning and execution. SS, TAL and RKS participated in discussion. All authors read and approved the manuscript.

## Data Availability

The data that support the findings of this study are available from the corresponding author upon reasonable request.
